# Potassium l-2-nitrimino-1,3-diazepane-4-carboxyl­ate monohydrate

**DOI:** 10.1107/S1600536808023817

**Published:** 2008-10-09

**Authors:** Harutyun A. Karapetyan

**Affiliations:** a Molecule Structure Research Center, National Academy of Sciences RA, Azatutyan Avenue 26, 375014 Yerevan, Republic of Armenia

## Abstract

The title compound, K^+^·C_6_H_9_N_4_O_4_^−^·H_2_O, crystallizes with the K atoms located on special positions related by pseudocentres of symmetry. Each K atom is coordinated by six O-atom donors. The N and water H atoms are involved in inter- and intra­molecular N—H⋯O, N—H⋯N and O—H⋯O hydrogen bonding. The data indicate inversion twinning.

## Related literature

For related literature, see: Apreyan & Petrosyan (2008 ▶); Apreyan *et al.* (2008*a* ▶,*b* ▶); Karapetyan (2008*a* ▶,*b* ▶); Karapetyan *et al.* (2007 ▶); Kurtz & Perry (1968 ▶); Paul *et al.* (1961 ▶); Petrosyan *et al.* (2005 ▶).
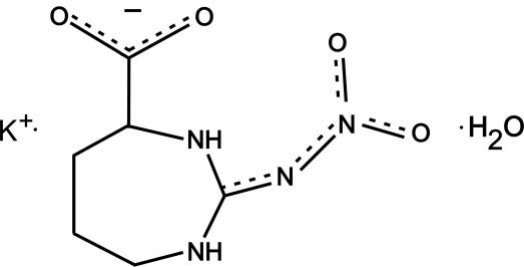


## Experimental

### 

#### Crystal data

K^+^·C_6_H_9_N_4_O_4_^−^·H_2_O
*M* *_r_* = 258.29
Orthorhombic, 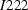

*a* = 7.3883 (15) Å
*b* = 10.087 (2) Å
*c* = 29.031 (6) Å
*V* = 2163.5 (8) Å^3^
*Z* = 8
Mo *K*α radiation
μ = 0.51 mm^−1^
*T* = 293 (2) K
0.21 × 0.14 × 0.11 mm

#### Data collection

Enraf–Nonius CAD-4 diffractometer
Absorption correction: none
5030 measured reflections
3141 independent reflections
1736 reflections with *I* > 2σ(*I*)
*R*_int_ = 0.030
3 standard reflections every 400 reflections intensity decay: none

#### Refinement

*R*[*F* ^2^ > 2σ(*F* ^2^)] = 0.065
*wR*(*F* ^2^) = 0.193
*S* = 1.04
3141 reflections
154 parameters
3 restraints
H atoms treated by a mixture of independent and constrained refinement
Δρ_max_ = 0.42 e Å^−3^
Δρ_min_ = −0.39 e Å^−3^
Absolute structure: Flack (1983 ▶), 1350 Friedel pairs
Flack parameter: 0.48 (20)

### 

Data collection: *DATCOL* in *CAD-4 Software* (Enraf–Nonius, 1988 ▶); cell refinement: *LS* in *CAD-4 Software*; data reduction: *HELENA* (Spek, 1997 ▶); program(s) used to solve structure: *SHELXS97* (Sheldrick, 2008 ▶); program(s) used to refine structure: *SHELXL97* (Sheldrick, 2008 ▶); molecular graphics: *SHELXTL* (Sheldrick, 2008 ▶); software used to prepare material for publication: *SHELXTL*.

## Supplementary Material

Crystal structure: contains datablocks global, I. DOI: 10.1107/S1600536808023817/hg2410sup1.cif

Structure factors: contains datablocks I. DOI: 10.1107/S1600536808023817/hg2410Isup2.hkl

Additional supplementary materials:  crystallographic information; 3D view; checkCIF report

## Figures and Tables

**Table 1:** Hydrogen-bond geometry (Å, °)

*D*—H⋯*A*	*D*—H	H⋯*A*	*D*⋯*A*	*D*—H⋯*A*
O5—H11⋯O1^i^	0.87 (2)	2.09 (5)	2.885 (5)	153 (10)
O5—H10⋯O3^ii^	0.856 (19)	2.03 (4)	2.793 (4)	148 (6)
N2—H9⋯N3^iii^	0.86	2.39	3.080 (4)	138
N1—H2⋯O3	0.86	2.09	2.561 (5)	114

Symmetry codes: (i) 
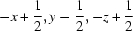
; (ii) 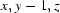; (iii) 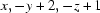.

**Fig. 1.**
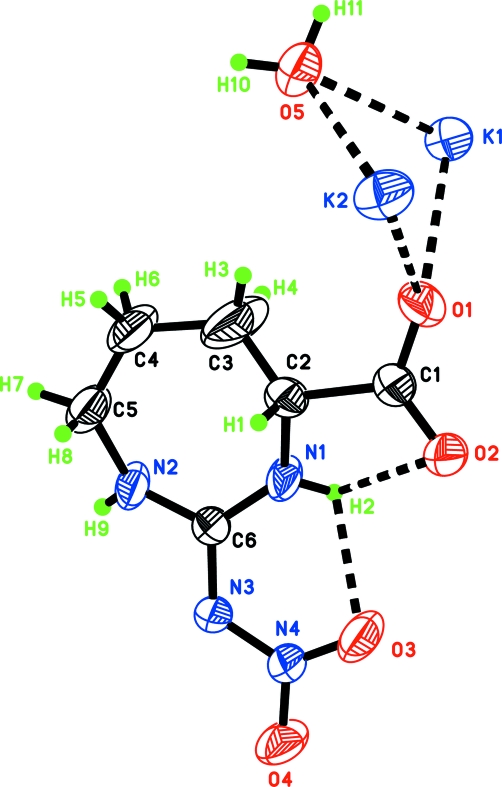
A perspective view of the asymmetric unit, showing the atomic numbering and displacement ellipsoids drawn at the 50% probability level.

**Fig. 2.**
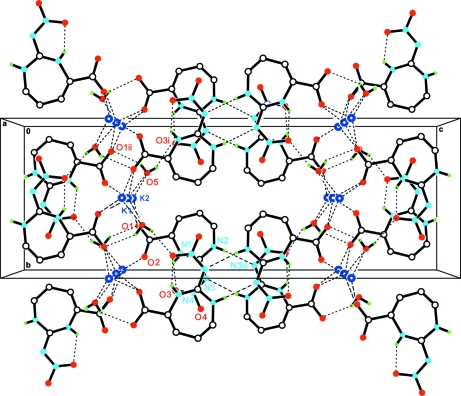
Packing of the molecules. For clarity, only the donors in the original molecule and their corresponding acceptors are labelled. Symmetry codes are: (i) x, y - 1, z; (ii)-x + 1/2, y - 1/2, -z + 1/2; (iii) x, 2 - y, 1 - z. Dashed lines indicate hydrogen bonds. H atoms not involved in hydrogen bonding have been omitted.

## supplementary crystallographic information

### Comment

Cyclic L-2-nitrimino-1,3-diazepane-4-carboxylic acid (L-NIDCA), produced by elimination of amine from L-nitroarginine (Paul *et al.*, 1961) , may generate new  non-linear optical materials like  L-nitroarginine itself [Apreyan *et al.* (2008*a*, 2008*b*); Karapetyan *et al.*(2007); Petrosyan *et al.* (2005); the crystal structures of L-NIDCA and its monohydrate have been recently reported [Karapetyan (2008a, 2008b)].

This paper presents a structural study of the potassium salt of L-NIDCA monohydrate.  The structure was solved and refined in the orthorhombic unit cell with I222 space group. The choice of the non-centric space group was based on the generation of second harmonic observed on a powder sample (YAG:Nd laser, Kurtz-Perry method [Kurtz & Perry,1968]). In this structure, two independent potassium cations occupy special positions. These potassium atoms are located about pseudo-inversion centers, which is most likely the reason for the presence of high level pseudosymmetry in the structure. Both potassium cations are coordinated by six oxygen atoms with K···O bond lengths  in the ranges 2.712 (5)-2.815 (7) Å for K1 and 2.642 (5)-2.783 (6) Å for K2.

A view of the asymmetric unit is shown in Fig. 1. The high value of U_eq_ of atom C3 of the 1,3-diazepane ring compared to those of its neighbors indicates potential disorder of this atom. In the crystal structure, the nitrogen-bound H atoms and the water H atoms are involved in N—H···O, N—H···N and O—H···O hydrogen bonding (Table 1), one of them being intra- and the other three intermolecular, linking anions and water molecules in infinite layers parallel to the *bc* plane (Fig. 2).

### Experimental

The title compound was synthesized from a mixture of aqueous solutions containing L-nitroarginine (2 g, Sigma-Aldrich) and KOH (0.512 g) at room temperature. Single crystals of the title compound were obtained by slow evaporation of the solution. At 97° C decomposition of the crystals was observed.

### Refinement

The data set was collected in a full sphere of reciprocal space. Space group I222 was chosen on the basis of the powder second harmonic of YAG:Nb laser generation property of the crystals of the title compound. In spite of the fact that all H atoms appear in difference Fourier maps in reasonable positions, they became unacceptable after refinement. Because of this, all the H atoms except those belonging to the water molecule were placed in geometrically calculated positions and included in the refinement in a riding model approximation, with *U*_iso_(H) = 1.2*U*_eq_(carrier atom). The positions of the H atoms of the water molecule were located in difference Fourier maps and included in the refinement with fixed O—H (0.85 Å), H···H (1.35 Å) distances and isotropic temperature parameters *U*_iso_(H) = 1.4*U*_eq_(O). The absolute configuration has been determined using L-nitroarginine of known absolute configuration.

### Crystal data

**Table:**

K^+^·C_6_H_9_N_4_O_4_^−^·H_2_O	*F*(000) = 1072
*M**_r_* = 258.29	*D*_x_ = 1.586 Mg m^−^^3^
Orthorhombic, *I*222	Mo *K*α radiation, λ = 0.71073 Å
Hall symbol: I 2 2	Cell parameters from 24 reflections
*a* = 7.3883 (15) Å	θ = 14–16°
*b* = 10.087 (2) Å	µ = 0.51 mm^−^^1^
*c* = 29.031 (6) Å	*T* = 293 K
*V* = 2163.5 (8) Å^3^	Prism, yellow
*Z* = 8	0.21 × 0.14 × 0.11 mm

### Data collection

**Table:**

Enraf–Nonius CAD-4 diffractometer	*R*_int_ = 0.030
Radiation source: fine-focus sealed tube	θ_max_ = 30.0°, θ_min_ = 2.1°
graphite	*h* = −10→10
ω/2θ scans	*k* = −13→13
5030 measured reflections	*l* = −38→38
3141 independent reflections	3 standard reflections every 400 reflections
1726 reflections with *I* > 2σ(*I*)	intensity decay: none

### Refinement

**Table:**

Refinement on *F*^2^	Secondary atom site location: difference Fourier map
Least-squares matrix: full	Hydrogen site location: inferred from neighbouring sites
*R*[*F*^2^ > 2σ(*F*^2^)] = 0.065	H atoms treated by a mixture of independent and constrained refinement
*wR*(*F*^2^) = 0.193	*w* = 1/[σ^2^(*F*_o_^2^) + (0.0676*P*)^2^ + 6.1136*P*] where *P* = (*F*_o_^2^ + 2*F*_c_^2^)/3
*S* = 1.04	(Δ/σ)_max_ < 0.001
3141 reflections	Δρ_max_ = 0.43 e Å^−^^3^
154 parameters	Δρ_min_ = −0.39 e Å^−^^3^
3 restraints	Absolute structure: Flack (1983), 1350 Friedel pairs
Primary atom site location: structure-invariant direct methods	Flack parameter: 0.48 (20)

### Special details

**Table:**

Geometry. All esds (except the esd in the dihedral angle between two l.s. planes) are estimated using the full covariance matrix. The cell esds are taken into account individually in the estimation of esds in distances, angles and torsion angles; correlations between esds in cell parameters are only used when they are defined by crystal symmetry. An approximate (isotropic) treatment of cell esds is used for estimating esds involving l.s. planes.
Refinement. Refinement of F^2^ against ALL reflections. The weighted *R*-factor *wR* and goodness of fit *S* are based on F^2^, conventional *R*-factors *R* are based on *F*, with *F* set to zero for negative *F*^2^. The threshold expression of *F*^2^ > σ(*F*^2^) is used only for calculating *R*-factors(gt) *etc*. and is not relevant to the choice of reflections for refinement. *R*-factors based on *F*^2^ are statistically about twice as large as those based on *F*, and *R*- factors based on ALL data will be even larger.

### Fractional atomic coordinates and isotropic or equivalent isotropic displacement parameters (Å^2^)

**Table:**

	*x*	*y*	*z*	*U*_iso_*/*U*_eq_	
K1	0.5000	0.5000	0.26313 (7)	0.0481 (5)	
K2	0.0000	0.5000	0.26268 (8)	0.0536 (5)	
O1	0.2429 (10)	0.6833 (3)	0.28276 (9)	0.0510 (8)	
O2	0.2513 (10)	0.8993 (3)	0.29658 (9)	0.0618 (10)	
O3	0.2476 (15)	1.1286 (3)	0.37039 (10)	0.0905 (16)	
O4	0.2389 (10)	1.2557 (3)	0.42935 (11)	0.0723 (12)	
O5	0.2461 (12)	0.3267 (3)	0.30317 (11)	0.0644 (10)	
N1	0.2755 (14)	0.8784 (3)	0.38382 (11)	0.0630 (19)	
H2	0.3373	0.9320	0.3670	0.076*	
N2	0.2659 (10)	0.8275 (3)	0.46137 (11)	0.0583 (14)	
H9	0.3224	0.8526	0.4858	0.070*	
N3	0.2516 (11)	1.0441 (3)	0.44244 (10)	0.0490 (10)	
N4	0.2478 (12)	1.1416 (3)	0.41290 (11)	0.0526 (10)	
C1	0.2419 (12)	0.7820 (4)	0.30906 (13)	0.0433 (11)	
C2	0.2101 (7)	0.7544 (4)	0.36059 (14)	0.0397 (12)	
H1	0.0794	0.7470	0.3657	0.048*	
C3	0.294 (2)	0.6381 (6)	0.37834 (19)	0.118 (5)	
H4	0.4240	0.6514	0.3793	0.142*	
H3	0.2697	0.5645	0.3578	0.142*	
C4	0.2262 (15)	0.6022 (4)	0.42740 (17)	0.069 (2)	
H5	0.1150	0.5526	0.4232	0.082*	
H6	0.3139	0.5404	0.4399	0.082*	
C5	0.1931 (9)	0.6894 (5)	0.46054 (17)	0.0601 (19)	
H7	0.2319	0.6481	0.4891	0.072*	
H8	0.0624	0.6974	0.4624	0.072*	
C6	0.2498 (15)	0.9152 (4)	0.42687 (13)	0.0475 (10)	
H10	0.288 (8)	0.279 (5)	0.3251 (13)	0.07 (2)*	
H11	0.288 (14)	0.287 (8)	0.2790 (13)	0.16 (5)*	

### Atomic displacement parameters (Å^2^)

**Table:**

	*U*^11^	*U*^22^	*U*^33^	*U*^12^	*U*^13^	*U*^23^
K1	0.0547 (12)	0.0388 (11)	0.0507 (11)	0.0026 (10)	0.000	0.000
K2	0.0566 (12)	0.0404 (12)	0.0637 (13)	−0.0021 (11)	0.000	0.000
O1	0.070 (2)	0.0423 (15)	0.0409 (14)	0.000 (3)	0.008 (3)	−0.0129 (13)
O2	0.115 (3)	0.0374 (15)	0.0326 (13)	0.013 (4)	−0.002 (4)	0.0002 (11)
O3	0.202 (5)	0.0350 (16)	0.0343 (15)	−0.001 (5)	−0.009 (5)	0.0075 (12)
O4	0.136 (4)	0.0279 (13)	0.0534 (18)	0.006 (4)	−0.007 (4)	−0.0024 (13)
O5	0.103 (3)	0.0470 (17)	0.0436 (17)	−0.009 (4)	−0.003 (4)	0.0143 (15)
N1	0.134 (6)	0.0286 (16)	0.0266 (16)	−0.021 (4)	0.012 (3)	−0.0014 (12)
N2	0.115 (4)	0.0309 (16)	0.0291 (15)	−0.017 (3)	0.012 (4)	−0.0006 (13)
N3	0.087 (3)	0.0286 (14)	0.0312 (15)	0.007 (4)	0.006 (4)	0.0001 (12)
N4	0.088 (3)	0.0310 (16)	0.0393 (17)	0.005 (4)	0.014 (4)	0.0017 (13)
C1	0.057 (3)	0.039 (2)	0.0339 (18)	0.007 (4)	−0.010 (3)	−0.0022 (15)
C2	0.049 (3)	0.0352 (19)	0.0344 (19)	0.004 (2)	−0.002 (2)	−0.0046 (16)
C3	0.267 (17)	0.043 (3)	0.043 (3)	0.046 (7)	−0.002 (6)	0.008 (2)
C4	0.125 (6)	0.029 (2)	0.052 (2)	−0.013 (4)	−0.008 (5)	0.0091 (18)
C5	0.098 (6)	0.037 (2)	0.045 (3)	0.001 (3)	0.004 (3)	0.011 (2)
C6	0.079 (3)	0.0335 (17)	0.0298 (17)	−0.002 (5)	0.001 (5)	−0.0016 (14)

### Geometric parameters (Å, °)

**Table:**

K1—O1	2.712 (5)	O4—N4	1.247 (4)
K1—O1^i^	2.712 (5)	O5—H10	0.856 (19)
K1—O2^ii^	2.736 (6)	O5—H11	0.87 (2)
K1—O2^iii^	2.736 (6)	N1—C6	1.318 (5)
K1—O5^i^	2.815 (7)	N1—C2	1.502 (6)
K1—O5	2.815 (7)	N1—H2	0.8600
K1—C1^ii^	3.524 (5)	N2—C6	1.342 (5)
K1—C1^iii^	3.524 (5)	N2—C5	1.494 (7)
K1—K2	3.6942 (8)	N2—H9	0.8600
K1—K2^iv^	3.6942 (8)	N3—N4	1.305 (4)
K1—H11	2.70 (11)	N3—C6	1.377 (5)
K2—O1	2.642 (5)	C1—C2	1.540 (6)
K2—O1^v^	2.642 (5)	C1—K1^viii^	3.524 (5)
K2—O2^vi^	2.714 (6)	C2—C3	1.423 (9)
K2—O2^ii^	2.714 (6)	C2—H1	0.9800
K2—O5	2.783 (6)	C3—C4	1.552 (9)
K2—O5^v^	2.783 (6)	C3—H4	0.9700
K2—K1^vii^	3.6942 (8)	C3—H3	0.9700
K2—H11	3.06 (6)	C4—C5	1.326 (7)
O1—C1	1.255 (4)	C4—H5	0.9700
O2—C1	1.239 (5)	C4—H6	0.9700
O2—K2^viii^	2.714 (6)	C5—H7	0.9700
O2—K1^viii^	2.736 (6)	C5—H8	0.9700
O3—N4	1.241 (4)		
			
O1—K1—O1^i^	155.74 (15)	O2^ii^—K2—K1^vii^	132.78 (15)
O1—K1—O2^ii^	84.89 (16)	O5—K2—K1^vii^	130.69 (16)
O1^i^—K1—O2^ii^	110.81 (14)	O5^v^—K2—K1^vii^	49.08 (15)
O1—K1—O2^iii^	110.81 (14)	O1—K2—K1	47.16 (12)
O1^i^—K1—O2^iii^	84.89 (16)	O1^v^—K2—K1	132.72 (12)
O2^ii^—K1—O2^iii^	101.4 (2)	O2^vi^—K2—K1	132.78 (15)
O1—K1—O5^i^	87.51 (15)	O2^ii^—K2—K1	47.57 (13)
O1^i^—K1—O5^i^	82.52 (14)	O5—K2—K1	49.08 (15)
O2^ii^—K1—O5^i^	160.81 (11)	O5^v^—K2—K1	130.69 (16)
O2^iii^—K1—O5^i^	65.09 (14)	K1^vii^—K2—K1	179.60 (13)
O1—K1—O5	82.52 (14)	O1—K2—H11	89 (2)
O1^i^—K1—O5	87.51 (15)	O1^v^—K2—H11	87 (2)
O2^ii^—K1—O5	65.09 (14)	O2^vi^—K2—H11	146.2 (10)
O2^iii^—K1—O5	160.81 (11)	O2^ii^—K2—H11	50.6 (8)
O5^i^—K1—O5	131.23 (19)	O5—K2—H11	16.1 (6)
O1—K1—C1^ii^	101.25 (17)	O5^v^—K2—H11	146.1 (6)
O1^i^—K1—C1^ii^	93.15 (14)	K1^vii^—K2—H11	134 (2)
O2^ii^—K1—C1^ii^	17.69 (15)	K1—K2—H11	46 (2)
O2^iii^—K1—C1^ii^	101.48 (14)	C1—O1—K2	133.3 (6)
O5^i^—K1—C1^ii^	166.12 (17)	C1—O1—K1	132.3 (5)
O5—K1—C1^ii^	61.33 (12)	K2—O1—K1	87.26 (8)
O1—K1—C1^iii^	93.15 (14)	C1—O2—K2^viii^	125.5 (5)
O1^i^—K1—C1^iii^	101.25 (17)	C1—O2—K1^viii^	120.1 (6)
O2^ii^—K1—C1^iii^	101.48 (14)	K2^viii^—O2—K1^viii^	85.34 (8)
O2^iii^—K1—C1^iii^	17.69 (15)	K2—O5—K1	82.59 (8)
O5^i^—K1—C1^iii^	61.33 (12)	K2—O5—H10	155 (4)
O5—K1—C1^iii^	166.12 (17)	K1—O5—H10	114 (5)
C1^ii^—K1—C1^iii^	107.02 (17)	K2—O5—H11	101 (5)
O1—K1—K2	45.59 (12)	K1—O5—H11	74 (7)
O1^i^—K1—K2	134.53 (12)	H10—O5—H11	102 (3)
O2^ii^—K1—K2	47.09 (13)	C6—N1—C2	127.9 (6)
O2^iii^—K1—K2	132.56 (15)	C6—N1—H2	116.0
O5^i^—K1—K2	131.90 (15)	C2—N1—H2	116.0
O5—K1—K2	48.33 (15)	C6—N2—C5	124.8 (5)
C1^ii^—K1—K2	59.38 (14)	C6—N2—H9	117.6
C1^iii^—K1—K2	120.34 (15)	C5—N2—H9	117.6
O1—K1—K2^iv^	134.53 (12)	N4—N3—C6	119.7 (3)
O1^i^—K1—K2^iv^	45.59 (12)	O3—N4—O4	118.6 (3)
O2^ii^—K1—K2^iv^	132.56 (15)	O3—N4—N3	125.0 (3)
O2^iii^—K1—K2^iv^	47.09 (13)	O4—N4—N3	116.4 (3)
O5^i^—K1—K2^iv^	48.33 (15)	O2—C1—O1	125.4 (4)
O5—K1—K2^iv^	131.90 (15)	O2—C1—C2	117.8 (3)
C1^ii^—K1—K2^iv^	120.34 (15)	O1—C1—C2	116.6 (4)
C1^iii^—K1—K2^iv^	59.38 (14)	O2—C1—K1^viii^	42.2 (4)
K2—K1—K2^iv^	179.60 (13)	O1—C1—K1^viii^	97.8 (3)
O1—K1—H11	95.8 (12)	C2—C1—K1^viii^	127.8 (4)
O1^i^—K1—H11	80.1 (15)	C3—C2—N1	112.6 (5)
O2^ii^—K1—H11	54.4 (13)	C3—C2—C1	115.8 (5)
O2^iii^—K1—H11	142.9 (4)	N1—C2—C1	103.6 (4)
O5^i^—K1—H11	144.2 (12)	C3—C2—H1	108.2
O5—K1—H11	17.9 (4)	N1—C2—H1	108.2
C1^ii^—K1—H11	46.4 (10)	C1—C2—H1	108.2
C1^iii^—K1—H11	153.2 (8)	C2—C3—C4	112.6 (8)
K2—K1—H11	54.5 (15)	C2—C3—H4	109.1
K2^iv^—K1—H11	125.5 (15)	C4—C3—H4	109.1
O1—K2—O1^v^	154.51 (16)	C2—C3—H3	109.1
O1—K2—O2^vi^	109.73 (14)	C4—C3—H3	109.1
O1^v^—K2—O2^vi^	86.68 (16)	H4—C3—H3	107.8
O1—K2—O2^ii^	86.68 (16)	C5—C4—C3	124.8 (4)
O1^v^—K2—O2^ii^	109.73 (14)	C5—C4—H5	106.1
O2^vi^—K2—O2^ii^	101.3 (2)	C3—C4—H5	106.1
O1—K2—O5	84.41 (15)	C5—C4—H6	106.1
O1^v^—K2—O5	84.89 (16)	C3—C4—H6	106.1
O2^vi^—K2—O5	160.93 (12)	H5—C4—H6	106.3
O2^ii^—K2—O5	65.81 (15)	C4—C5—N2	124.3 (5)
O1—K2—O5^v^	84.89 (16)	C4—C5—H7	106.3
O1^v^—K2—O5^v^	84.41 (15)	N2—C5—H7	106.3
O2^vi^—K2—O5^v^	65.81 (15)	C4—C5—H8	106.3
O2^ii^—K2—O5^v^	160.93 (12)	N2—C5—H8	106.3
O5—K2—O5^v^	130.0 (2)	H7—C5—H8	106.4
O1—K2—K1^vii^	132.72 (12)	N1—C6—N2	120.6 (4)
O1^v^—K2—K1^vii^	47.16 (12)	N1—C6—N3	125.2 (4)
O2^vi^—K2—K1^vii^	47.57 (13)	N2—C6—N3	112.1 (3)
			
O1^v^—K2—O1—C1	−49.9 (4)	O2^ii^—K1—O5—K2	−53.73 (14)
O2^vi^—K2—O1—C1	77.6 (4)	O2^iii^—K1—O5—K2	−101.4 (5)
O2^ii^—K2—O1—C1	178.5 (4)	O5^i^—K1—O5—K2	114.12 (11)
O5—K2—O1—C1	−115.5 (4)	C1^ii^—K1—O5—K2	−73.12 (16)
O5^v^—K2—O1—C1	15.6 (4)	C1^iii^—K1—O5—K2	−38.4 (6)
K1^vii^—K2—O1—C1	27.7 (5)	K2^iv^—K1—O5—K2	−179.55 (14)
K1—K2—O1—C1	−151.7 (4)	C6—N3—N4—O3	−2.1 (16)
O1^v^—K2—O1—K1	101.82 (8)	C6—N3—N4—O4	175.9 (10)
O2^vi^—K2—O1—K1	−130.65 (16)	K2^viii^—O2—C1—O1	−49.8 (12)
O2^ii^—K2—O1—K1	−29.78 (10)	K1^viii^—O2—C1—O1	57.6 (12)
O5—K2—O1—K1	36.21 (12)	K2^viii^—O2—C1—C2	135.8 (5)
O5^v^—K2—O1—K1	167.33 (12)	K1^viii^—O2—C1—C2	−116.8 (5)
K1^vii^—K2—O1—K1	179.47 (16)	K2^viii^—O2—C1—K1^viii^	−107.4 (3)
O1^i^—K1—O1—C1	49.8 (4)	K2—O1—C1—O2	−114.3 (9)
O2^ii^—K1—O1—C1	−178.2 (4)	K1—O1—C1—O2	105.4 (9)
O2^iii^—K1—O1—C1	−77.9 (4)	K2—O1—C1—C2	60.2 (8)
O5^i^—K1—O1—C1	−15.8 (4)	K1—O1—C1—C2	−80.1 (8)
O5—K1—O1—C1	116.3 (4)	K2—O1—C1—K1^viii^	−79.4 (3)
C1^ii^—K1—O1—C1	175.0 (4)	K1—O1—C1—K1^viii^	140.3 (2)
C1^iii^—K1—O1—C1	−76.9 (5)	C6—N1—C2—C3	−67.0 (11)
K2—K1—O1—C1	152.2 (4)	C6—N1—C2—C1	167.2 (9)
K2^iv^—K1—O1—C1	−28.3 (5)	O2—C1—C2—C3	−146.7 (9)
O1^i^—K1—O1—K2	−102.45 (8)	O1—C1—C2—C3	38.4 (11)
O2^ii^—K1—O1—K2	29.60 (10)	K1^viii^—C1—C2—C3	164.0 (6)
O2^iii^—K1—O1—K2	129.84 (15)	O2—C1—C2—N1	−22.9 (10)
O5^i^—K1—O1—K2	−168.04 (12)	O1—C1—C2—N1	162.2 (8)
O5—K1—O1—K2	−35.89 (12)	K1^viii^—C1—C2—N1	−72.2 (6)
C1^ii^—K1—O1—K2	22.82 (11)	N1—C2—C3—C4	72.2 (10)
C1^iii^—K1—O1—K2	130.87 (14)	C1—C2—C3—C4	−168.9 (7)
K2^iv^—K1—O1—K2	179.46 (17)	C2—C3—C4—C5	−39.8 (16)
O1—K2—O5—K1	−34.97 (11)	C3—C4—C5—N2	−19.3 (15)
O1^v^—K2—O5—K1	168.20 (12)	C6—N2—C5—C4	52.9 (12)
O2^vi^—K2—O5—K1	104.1 (5)	C2—N1—C6—N2	42.7 (16)
O2^ii^—K2—O5—K1	53.89 (13)	C2—N1—C6—N3	−154.9 (9)
O5^v^—K2—O5—K1	−113.49 (10)	C5—N2—C6—N1	−43.0 (15)
K1^vii^—K2—O5—K1	−179.56 (14)	C5—N2—C6—N3	152.5 (8)
O1—K1—O5—K2	34.09 (11)	N4—N3—C6—N1	12.3 (17)
O1^i^—K1—O5—K2	−168.08 (12)	N4—N3—C6—N2	176.0 (9)

Symmetry codes: (i) −*x*+1, −*y*+1, *z*; (ii) −*x*+1/2, *y*−1/2, −*z*+1/2; (iii) *x*+1/2, −*y*+3/2, −*z*+1/2; (iv) *x*+1, *y*, *z*; (v) −*x*, −*y*+1, *z*; (vi) *x*−1/2, −*y*+3/2, −*z*+1/2; (vii) *x*−1, *y*, *z*; (viii) −*x*+1/2, *y*+1/2, −*z*+1/2.

### Hydrogen-bond geometry (Å, °)

**Table:**

*D*—H···*A*	*D*—H	H···*A*	*D*···*A*	*D*—H···*A*
O5—H11···O1^ii^	0.87 (2)	2.09 (5)	2.885 (5)	153 (10)
O5—H10···O3^ix^	0.86 (2)	2.03 (4)	2.793 (4)	148 (6)
N2—H9···N3^x^	0.86	2.39	3.080 (4)	138
N1—H2···O3	0.86	2.09	2.561 (5)	114

Symmetry codes: (ii) −*x*+1/2, *y*−1/2, −*z*+1/2; (ix) *x*, *y*−1, *z*; (x) *x*, −*y*+2, −*z*+1.
